# Neoadjuvant chemotherapy in combination with surgery in the treatment of local advanced breast cancer

**DOI:** 10.12669/pjms.35.5.310

**Published:** 2019

**Authors:** Haixia Zhao, Jinying Zhang, Yanxia Lu, Jihai Jin

**Affiliations:** 1 Haixia Zhao Department of General Surgery C (Breast Surgery), Binzhou People’s Hospital, Shandong, 256610, China; 2 Jinying Zhang Department of Cardio-Thoracic Surgery B, Binzhou people’s Hospital, 256603, China; 3 Yanxia Lu Department of Critical Care Medicine, Binzhou people’s Hospital, 256603, China; 4 Jihai Jin Department of General Surgery C (Breast Surgery), Binzhou People’s Hospital, Shandong, 256610, China

**Keywords:** Neoadjuvant chemotherapy, Locally advanced breast cancer, Modified radical mastectomy

## Abstract

**Objective:**

To investigate the effect of neoadjuvant chemotherapy combined with surgery on locally advanced breast cancer and its prognosis.

**Methods:**

One hundred and fifty-four patients with locally advanced breast cancer who were admitted to our hospital from February 2014 to April 2015 were selected as the study subjects. They were divided into an observation group and a control group according to the principle of random equalization, 77 each group. The observation group was treated with TAC scheme, neoadjuvant chemotherapy combined with modified radical resection, and continuously treated with the same scheme after operation until the end of the course of treatment. The control group was treated with modified radical resection and TAC scheme. The clinical efficacy of the two groups was observed, and the perioperative indications, prognosis and occurrence of adverse reactions were compared between the two groups.

**Results:**

The total effective rate of the observation group was 76.62%, significantly higher than that of the control group (55.84%, P<0.05). The observation group had shorter operation time and hospitalization time and less bleeding amount compared to the control group (P<0.05). The metastasis rate and recurrence rate of the observation group were significantly lower than those of the control group (P<0.05); there was a significant difference between the two groups (P<0.05). The one-year and three-year survival rates of the observation group were significantly higher than those of the control group (P<0.05). There was no significant difference in the incidence of adverse reactions between the two groups after operation (P>0.05).

**Conclusion:**

Preoperative neoadjuvant chemotherapy in combination with TAC scheme can reduce the difficulty of operation, improve the curative effect of patients, significantly improve the prognosis of patients and prolong the survival time, which is worth clinical application.

## INTRODUCTION

Breast cancer, a clinically common female malignant tumor with an increasing incidence in recent years, has now ranked the first among all female malignant tumors.^1^,^2^ Currently, it is widely believed that breast cancer is a systemic disease that is highly prone to metastasis.^3^,^4^ Locally advanced breast cancer (LABC) mainly refers to breast cancer with diameter of primary tumor lesions more than 5 cm (T3), skin and chest wall adhesion (T4) or regional lymph node fusion (N2).^5^ Studies have shown that the proportion of LABC is relatively high in new-onset breast cancer,^6^,^7^ and even reached more than 25% in developing countries. Although LABC has no distant metastatic lesion, its primary tumor lesion is large usually; so it is mainly treated with modified radical mastectomy. However, postoperative tumors tend to remain on the chest wall and skin edge, which leads to a high recurrence rate.^8^ In recent years, the treatment of LABC has made great progress with the development of neoadjuvant chemotherapy, and a study showed that the use of neoadjuvant chemotherapy before surgery could effectively reduce tumor lesions.^9^ Neoadjuvant chemotherapy can degrade the clinical stages for patients, improve the surgical resection rate, facilitate the clarification of the sensitivity of chemotherapy drugs to treat tumors, and control the potential micrometastasis to prevent distant metastasis.^10^,^11^ However, the current selection of therapeutic schemes and treatment cycle for neoadjuvant chemotherapy remain controversial. This study explored the application and safety of TAC neoadjuvant chemotherapy combined with surgery in the treatment of LABC to provide a reference for the rational selection of neoadjuvant chemotherapy. The result is reported as follows.

## METHODS

A total of 154 patients with LABC who were admitted to our hospital from February 2014 to April 2015 were randomly divided into an observation group and a control group, 77 each. The ages of patients in the observation group varied from 34 to 75 years old, with an average age of (55.4±6.7) years old, and the tumors of 45 cases were on the left side and 32 cases on the right side. As for TNM clinical stages before chemotherapy, 30 cases were at stage IIb, 21 cases at stage IIIa, 17 cases at stage IIIb, and 9 cases at stage IIIc. As for biopsy pathological types, 62 cases were invasive ductal carcinoma, and 15 cases were invasive lobular carcinoma. The age of patients in the control group varied from 37 to 73 years old, with an average age of (55.8±6.2) years old, and the tumors of 47 cases were on the left side and 30 cases on the right side. As for TNM clinical stages before chemotherapy, 32 cases were at stage IIb, 23 cases at stage IIIa, 15 cases at stage IIIb and 7 cases at stage IIIc. As for biopsy pathological types, 58 cases were invasive ductal carcinoma, and 19 cases were invasive lobular carcinoma. There were no significant differences in the general data such as age and clinical stages between the two groups (P>0.05), indicating that the results were comparable. The study was approved by the ethics committee of the hospital, and all patients signed informed consent.

### Inclusion criteria:

(1) Patients confirmed with LABC by pathology, cytology and etc; (2) Patients who did not receive other chemotherapy before treatment; (3) Patients whose liver and kidney function, blood routine, abdominal B ultrasound, electrocardiogram, cardiac color doppler ultrasound and breast molybdenum target were examined before chemotherapy and had no distant metastasis.

### Exclusion Criteria:

(1) Patients who had contraindication to chemotherapy; (2) Patients who were allergic to chemotherapy drugs used in this study; (3) Patients who were expected to survive for less than 4 months; (4) Patients in lactation and gestation period; (5) Patients with abnormal blood coagulation and abnormal bone marrow function.

All patients received radiotherapy, and hormone receptor-positive patients were treated with endocrine therapy and personalized care. Before surgery, patients were informed with the common knowledge, treatment and nursing of breast cancer, and were given one-on-one psychological counseling to help them ease the stress, anxiety and other negative emotions caused by surgery, and to optimize the patient’s surgical treatment. The patient’s family was informed with the common knowledge so that the way they took care of the patient was more correct. After patients underwent breast cancer resection, their body hormone secretions were measured, postoperative medications were adjusted according to the different hormone secretion, and doses were rationalized according to the patients’ ages, eating habits, and whether or not they had menopause. Each patient’s personality varied, and the environment they required was also different. Firstly, it was necessary to ensure that the environment in which patients lived was relatively quiet, and was suitable for patients to improve immunity. Secondly, the living room of each patient was arranged according to his requirement.

### Observation group

Neoadjuvant chemotherapy was administered after the patients were confirmed with LABC and contraindications were excluded. The treatment was as follows. On the 1st day, each patient was intravenously infused with docetaxel at a dose of 75 mg/m^2^ (Haizheng Pfizer Pharmaceutical Co., Ltd, China; SFDA approval number: H20093520), cyclophosphamide at a dose of 600 mg/m^2^ (Shandong Xinshidai Pharmaceutical Co., Ltd., China; SFDA approval number: H20093392), and pirarubicin at a dose of 50 mg/m^2^ (Haizhenghuirui Pharmaceutical Co., Ltd., China; SFDA approval number: H20045983) for three hours. Three weeks was regarded as one treatment cycle, and 2 or 3 cycles were needed. In the course of chemotherapy, blood routine and liver, kidney, heart and other organ functions were closely monitored. When chemotherapy associated side effects occurred, timely symptomatic treatment was conducted. Within two weeks after the end of chemotherapy, patients underwent modified radical mastectomy. TAC scheme adjuvant chemotherapy continued after patients’ condition recovered and contraindications were excluded.

### Control group

After patients were confirmed with no contraindications, modified radical mastectomy was performed, and TAC scheme chemotherapy which was the same with the observation group was given after surgery. The blood routine indicators and functions of organs such as the liver, kidney, and heart were closely observed during chemotherapy. Timely symptomatic treatment was conducted when chemotherapy associated side effects occurred.

According to response evaluation criteria in solid tumors of World Healthcare Organization,^12^ there were four levels, complete remission (CR), tumor lesions completely disappeared after chemotherapy, partial remission (PR), primary lesion was reduced by more than 50% after chemotherapy, stable disease (SD), the tumor reduced by less than 50% or increased by less than 25%, and no new lesions appeared, and progressive disease (PD), tumor volume increased by over 25% or new lesions appeared. The computational formula of overall effective rate was overall effective rate = (number of CR cases + number of PR cases) / total number of cases × 100%.

Indicators such as surgery duration, intraoperative blood loss, intravascular tumor thrombus and intraoperative skin resection were compared between the two groups.

All patients were followed up for 26-36 months regularly after chemotherapy, and complications, tumor recurrence rate, metastasis rate and survival rate were compared.

### Statistical analysis

Data were analyzed by SPSS 20.0. Measurement data was represented by Mean±SD and processed by t test; enumeration data were expressed by (%) and processed by Chi-square test. P<0.05 meant that difference was statistically significant.

## RESULTS

**Fig.1:**
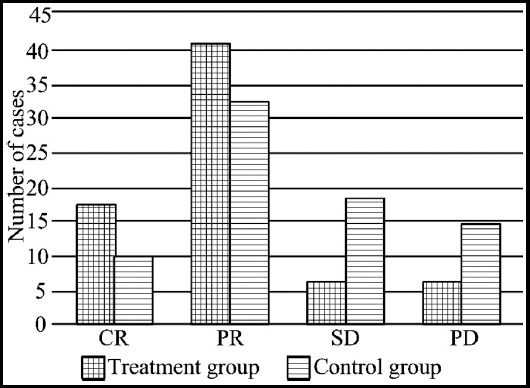
Comparison of clinical effects between the two groups.

The overall effective rate of the observation group was 76.62% (59/77), which was significantly higher than that of the control group (55.84% (44/77)). The difference was statistically significant (X^2^=8.781, P<0.05, Fig.1).

The surgery duration, intraoperative blood loss and postoperative hospital stay in the observation group were compared with those of the control group. The difference was statistically significant (P<0.05, Table-I).

**Table I:** Comparison of perioperative indications between the two groups.

Groups	Observation Group (n=77)	Control Group (n=77)	t	P
Surgery duration (min)	43.88±9.03	58.42±12.32	18.263	<0.05
Intraoperative blood loss (mL)	129.83±14.77	216.73±22.58	14.662	<0.05
Hospitalization duration (d)	9.25±1.28	13.33±2.38	6.589	<0.05

The metastasis rate and recurrence rate of breast cancer in the observation group were lower than those in the control group, and the differences were statistically significant (P<0.05, Table-II).

**Table II:** Comparison of postoperative tumor recurrence and metastasis rate between the two groups.

Groups		Observation group (n=77)	Control group (n=77)	X^2^	P
Metastasis	Bone Metastasis	6	13		
	Pulmonary metastasis	4	12		
Metastasis rate (%)		12.99	32.47	4.586	<0.05
Local recurrence		6	17		
Recurrence rate (%)		7.79	22.08	2.817	<0.05

The one-year and three-year survival rates of the observation group were significantly higher than those of the control group (P<0.05, Table-III).

**Table III:** Comparison of postoperative survival rates between the two groups.

Groups	Observation group (n=77)	Control group (n=77)	X^2^	P
One year after surgery	64 (83.12%)	48 (62.34%)	4.014	<0.05
Three years after surgery	54 (70.13%)	31 (40.26%)	8.586	<0.05

There were 11 cases of grade I to III gastrointestinal reaction (14.29%), 4 cases of grade IV myelosuppression (5.19%), 26 cases of alopecia (33.77%), and 17 cases of grade II to III leukopenia and neutropenia (22.08%) during the treatment in the observation group. There were 13 cases of grade I to III gastrointestinal reactions (16.88%), 6 cases of grade IV myelosuppression (7.79%), 30 cases of alopecia (38.96%), and 21 cases of grade II to III leukopenia and neutropenia reduction (27.27%). There was no significant difference in the incidence of gastrointestinal reactions, myelosuppression, alopecia, leukopenia and neutropenia between the two groups (X^2^=0.11, 0.00, 0.24, 0.30, P<0.05).

## DISCUSSION

For patients with LABC, the primary tumor lesions are usually large, and the effect of surgical treatment is not ideal. Some patients are easily influenced by the skin invasion, tumor chest wall fixation, axillary lymph node fusion and other factors, resulting in the inability to surgery and severely affecting the prognosis.^13^,^14^ The treatment of LABC has made great progress with the development and promotion of neo adjuvant chemotherapy, and neo adjuvant chemotherapy mainly aims to minimize the volume of tumors and reduce the preoperative clinical stages of breast cancer by administering chemotherapy to patients before surgery so that favorable conditions for surgery can be created and the surgical results can be improved.^15^,^16^ Besides, the drug regimen in neo adjuvant chemotherapy is not accurately clinically defined, while anthracyclines, docetaxel and cyclophosphamide are commonly used as neo adjuvant chemotherapy drugs. Jeon et al. reported that the application of neo adjuvant chemotherapy in the treatment of LABC could degrade the clinical stages to benefit surgery and clearly understand the sensitivity of breast cancer to chemotherapy drugs.^17^

The chemotherapy scheme used in this study was the TAC scheme (cyclophosphamide + docetaxel + pirarubicin). Cyclophosphamide is a cell cycle nonspecific agent.^18^ After entering the body, it splits and releases chloroethylphosphoramide with strong alkylation to inhibit tumor cells, but its immunosuppressive effects are more significant. Docetaxel is a new generation of taxane chemotherapeutic drug.^19^ Combined with free tubulin, tubulin can form stable microtubules to inhibits microtubule depolymerization, ultimately inhibiting the mitosis and proliferation of cells, and the drug also has certain effects of inducing apoptosis of tumor cells. Pirarubicin is an anthracycline cell cycle non-specific anti-tumor drug that can be directly inserted between DNA double strands to inhibits DNA polymerase,^20^ prevent nucleic acid synthesis, and prevent cells from dividing in G2 phase, finally leading tumor cell to death. It is also effective for those who are resistant to doxorubicin. A previous study has shown that neo adjuvant chemotherapy alone had a clinical remission rate of 56% in the treatment of LABC,^21^ and neo adjuvant chemotherapy based on anthracyclines combined with paclitaxelcan improved clinical remission rate by about 6% ~ 16%.^22^ The effective rate of neo adjuvant chemotherapy with TAC scheme in this study group was 76.62%, similar to the above results. Some studies showed that for the patients at stage III breast cancer who underwent neo adjuvant chemotherapy,^23^ the local recurrence rate was 5.1% within two years after surgery, while for those who did not undergo neo adjuvant chemotherapy, the local recurrence rate increased to 17.3% within 2 years after surgery. The local recurrence rate of the observation group was 7.79%, and the local recurrence rate of the control group was 22.08%, which was equivalent to the above results. The results of this study also showed that the survival rate of patients in the observation group was significantly higher than that of the control group, which was consistent with the results of Niu.^24^ His research suggested that neo adjuvant chemotherapy combined with surgical treatment could improve clinical efficacy, reduce the post-recurrence rate and metastasis rate, and prolong the survival time of patients, which reflected the significant value of this treatment.

It was pointed out that modified radical mastectomy had the disadvantages of large trauma, hemorrhage and slow recovery, and it might cause breast loss and affect aesthetics and quality of life.^25^ The surgery duration, hospitalization duration and intraoperative blood loss of the observation group were compared with those of the control group, and the difference was statistically significant (P<0.05), and it showed that surgery based on neoc adjuvant chemotherapy could significantly shorten the surgery time and hospital stay time, and reduce the intraoperative blood loss. In addition, this study also found that in the course of chemotherapy, patients in the two groups had common toxic and side effects including leukopenia and neutropenia, gastrointestinal reactions, myelosuppression, hair loss, etc., and gastrointestinal reactions and hair loss were mild. The difference in the incidence between the two groups was not obvious, so it is believed that the neoadjuvant chemotherapy is safe.

## CONCLUSION

The significance of neo adjuvant chemotherapy in the treatment of LABC has been deeply rooted in the hearts of the people. Neoadjuvant chemotherapy based on anthracycline combined with vaginal drugs or sequential scheme can benefit most patients with LABC, and the chemotherapy associated side effects are low and well tolerated, which is worth clinical application. However, as the study was single-center and had a small sample size, lymph node metastasis and molecular subtyping in the two groups were not considered; therefore further analysis is required in the future.

### Authors’ Contribution

**HXZ & JYZ:** Study design, data collection and analysis.

**HXZ, YXL & JHJ:** Manuscript preparation, drafting and revising.

**HXZ & JHJ:** Review and final approval of manuscript.
